# Single-cell RNA sequencing unravels mosquito biology and host–pathogen interactions

**DOI:** 10.1186/s13071-026-07460-8

**Published:** 2026-10-02

**Authors:** Li-Bo Liu, Hao-Bo Ye, Zu-Hang Tian, Xiao-Hui Zeng, Jia-Hong Wu

**Affiliations:** 1 Guizhou Key Laboratory of Microbio and Infectious Disease Prevention and Control, School of Basic Medicine, Guizhou Medical University, Guiyang, 561113 Guizhou China; 2 The Key and Characteristic Laboratory of Modern Pathogen Biology, School of Basic Medicine, Guizhou Medical University, Guiyang, 561113 Guizhou China; 3 Institute of Virology, School of Basic Medicine, Guizhou Medical University, Guiyang, 561113 Guizhou China

**Keywords:** Single-cell RNA sequencing, Mosquito, Host–pathogen interactions, Cell atlas, Vector control, Mosquito-borne disease

## Abstract

**Abstract:**

Single-cell RNA sequencing (scRNA-seq) is fundamentally reshaping the research paradigm of mosquito biology and host–pathogen interactions via its high-resolution transcriptomic profiling capability. This review first outlines the technical workflow and data analysis strategies of scRNA-seq, then highlights key applications of scRNA-seq in constructing mosquito cell atlases, deciphering post-blood-meal transcriptional remodeling, sexual dimorphism, and receptor coexpression patterns. Furthermore, the role of scRNA-seq in revealing the cellular tropism of mosquito-borne pathogens, host immune responses, and the identification of potential intervention targets is discussed. Despite current challenges such as the lack of specific cell markers and insufficient functional validation, scRNA-seq has provided a novel perspective for understanding mosquito biology at the single-cell level and laid an important foundation for developing targeted vector control strategies. Looking forward, integration with spatial transcriptomics, multiomics approaches, and dynamic experimental validation will further advance this field toward mechanistic research and translational applications.

**Graphical abstract:**

**Figure:**
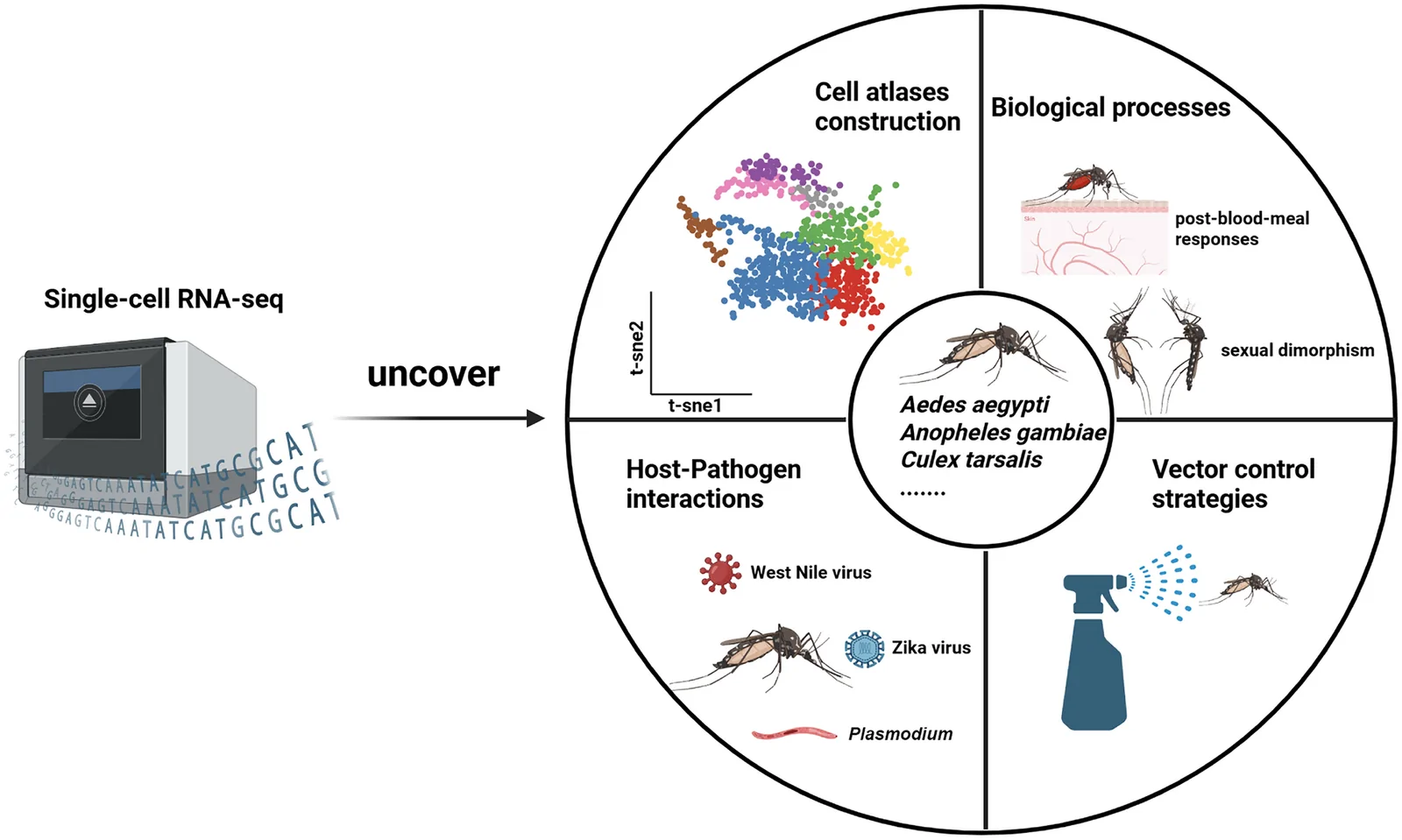

## Background

The transcriptome, encompassing the complete set of RNA molecules in cells or tissues, serves as the critical bridge linking the static genome with dynamic biological functions. Transcriptomic sequencing not only enables the identification of novel genes and alternative splicing events but also provides deep insights into fundamental processes such as development, differentiation, immune responses, and disease progression [1]. The advent of high-throughput sequencing has made transcriptome sequencing more cost-effective and efficient, thereby facilitating the advancement of transcriptome toward single-cell resolution [2]. In contrast to bulk transcriptome, the emerging technology of single-cell RNA sequencing (scRNA-seq) allows gene expression changes to be examined at the single-cell level [3, 4]. scRNA-seq not only overcomes the limitation of bulk transcriptome in masking cellular heterogeneity but also enables the discovery of rare cell types, the dissection of transcriptional diversity, the reconstruction of developmental trajectories, and the mapping of intercellular communication networks—capabilities that collectively transform our approach to investigating biological systems [5, 6]. Currently, scRNA-seq has driven groundbreaking advances in multiple fields, including oncology, neuroscience, and virology [7–9].

Mosquito-borne diseases such as malaria, dengue fever, and chikungunya fever have posed a serious threat to global public health. According to World Health Organization estimates, there are approximately 350 million annual cases of mosquito-borne diseases, resulting in ~700,000 deaths, a burden aggravated by climate-driven vector range expansion [10–12]. Therefore, understanding mosquito biology and mosquito–pathogen interactions is essential for developing vector control strategies. Traditional approaches, relying on bulk tissue analysis or candidate gene studies, are unable to address these issues with precision and efficiency. The advent of scRNA‑seq has overcome these limitations by deconstructing mosquito tissues into individual cell types, enabling the investigation of previously unanswerable questions, providing mechanistic insights that may challenge existing paradigms, and revealing novel intervention targets [13].

At present, scRNA‑seq has been applied to major mosquito vectors such as *Aedes aegypti*, *Anopheles gambiae*, and *Culex tarsalis*. Current studies have established cell atlases of midgut [14], fat body [15], hemocytes [13], and nervous system [16], uncovering novel cell subpopulations such as fat-body-yolk cells and megacytes [13, 15], dissecting cellular tropism of Zika virus (ZIKV) [17], West Nile virus [18], and *Plasmodium* [19], identifying susceptible cell types and specific immune responses. Yet these efforts remain preliminary, constrained by incomplete tissue coverage (e.g., ovaries, compound eyes), reliance on *Drosophila* orthology for cell annotation, and limited functional validation. Therefore, there is an urgent need to fully harness the potential of scRNA-seq to reshape our understanding of mosquito biology and disease transmission dynamics.

Consequently, this review aims to evaluate how scRNA-seq is reshaping mosquito biology. We first outline the principles and workflow of scRNA-seq technology and then highlight key findings of major vector species. Finally, we discuss current challenges and anticipate future directions for scRNA-seq in advancing our understanding of mosquito-borne disease transmission.

## Overview of scRNA-seq technology

**Fig. 1:**
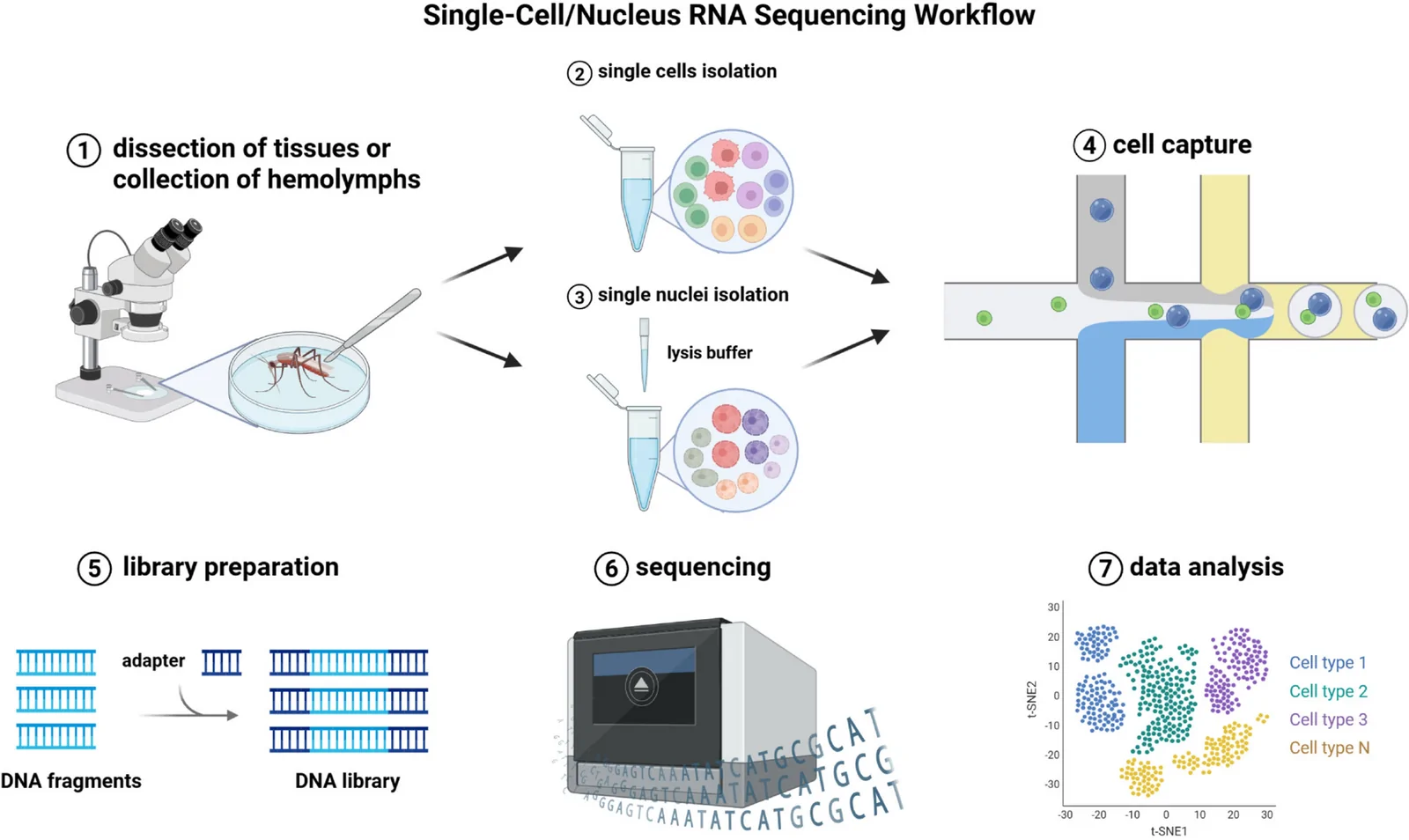
General workflow of single-cell/nucleus RNA sequencing in mosquitoes. (1) Fresh tissues are dissected, or hemolymphs are collected from mosquitoes. (2) Single cells are isolated using enzymatic or mechanical methods. (3) Nuclei are isolated using detergent‑based lysis and density gradient centrifugation. (4) Isolated cells/nuclei are captured using microfluidic platforms, microwell arrays, or FACS into multiwell plates. (5) Captured cells undergo reverse transcription to generate cDNA; after amplification, adapters are ligated to produce fragmented DNA libraries. (6) Libraries are sequenced on high‑throughput platforms to obtain transcriptome information. (7) Raw reads are mapped and processed via bioinformatic pipelines for quality control, clustering, and differential expression

The successful application of scRNA-seq in mosquitoes depends on a multistep workflow. A standard pipeline includes: (1) dissection of tissues or collection of hemolymphs from mosquitoes, (2) single cells/nuclei isolation, (3) cell capture, (4) library preparation, (5) high-throughput sequencing, and (6) data analysis (Fig. 1). Crucially, each step of scRNA-seq introduces key technical parameters that can profoundly influence the biological interpretation of the resulting data. Therefore, meticulous experimental design that matches these technical choices to the specific biological question is essential.

### Single-cell isolation, library construction, and sequencing

The initial and most critical challenge lies in obtaining a high-viability, unbiased suspension of single cells or nuclei from complex mosquito tissues. Dissociation protocols should be carefully optimized to minimize stress-induced transcriptional artifacts while maximizing cell yield and integrity. For tissues that are difficult to fully dissociate, such as the brain or fat body, single-nucleus RNA sequencing (snRNA-seq) provides an effective alternative [20]. In snRNA-seq, nuclei are isolated directly from frozen or fresh tissue without enzymatic dissociation, thereby preserving cell-type proportions and avoiding transcriptional stress responses induced by tissue processing. In contrast, scRNA-seq requires complete dissociation of tissues into individual cells, which can introduce dissociation-induced artifacts and selective loss of fragile or large cell types. However, researchers should be cognizant of the intrinsic limitations of snRNA-seq, including the loss of cytoplasmic transcripts and the potential underrepresentation of cell types with low nucleus-to-cytoplasm ratios. The choice between scRNA-seq and snRNA-seq is not merely technical but fundamentally shapes the cellular landscape that can be observed and interpreted in downstream analyses.

Following isolation, cells or nuclei are lysed to release RNA, which is then captured, reverse-transcribed, and amplified. The incorporation of unique molecular identifier (UMI) is a pivotal advancement in this workflow, allowing for the digital quantification of original transcripts and effectively mitigating polymerase chain reaction (PCR) amplification bias [21, 22]. The amplified products are typically fragmented, ligated with sequencing adapters to construct sequencing libraries, and ultimately sequenced on high-throughput platforms.

The evolution of scRNA-seq platforms over the past decade has largely focused on the balance between transcriptome depth and cellular throughput. Early methods based on micromanipulation or plate-based formats, such as the Tang method [23] and Smart-seq2 [24] offer near-complete transcript coverage per cell but are inherently limited in the number of cells profiled. In contrast, high-throughput droplet-based systems such as the 10× Genomics Chromium platform sacrifice full-length coverage for the ability to profile tens of thousands of cells, making them ideal for comprehensive cell atlas projects [25]. This fundamental trade-off has direct biological implications: droplet-based methods may miss low-abundance but functionally critical transcripts or fail to distinguish closely related cell subtypes, whereas plate-based methods might overlook rare cell populations owing to limited sampling. Emerging platforms continue to push these boundaries (Table 1). The choice of platform should be a deliberate decision, weighing the need to characterize rare cell types (favoring high throughput) against the need to resolve slight transcriptional changes or alternative splicing events (favoring high sensitivity and full-length reads).

**Table 1:** Summary of single-cell RNA sequencing technology

**Technology**	**Isolation strategies**	**Transcript coverage**	**Throughput**	**Amplification method**	**Sensitivity**	**Year**	**UMI**^**a**^	**References**
Smart-seq2	Plate	Full‐length	++^b^	PCR^c^	+++	2014	No	[24]
Smart-seq3	Plate	Full‐length	++	PCR	++++	2020	Yes	[26]
FLASH-seq	Plate	Full‐length	++	PCR	++++	2022	Yes	[27]
VASA-seq	Plate/droplet	Full‐length	+++	IVT^d^	++++	2022	Yes	[28]
inDrop-seq	Droplet	3′ end	+++	PCR	++	2015	Yes	[29]
Drop-seq	Droplet	3′ end	+++	PCR	++	2015	Yes	[30]
CEL-seq2	Plate	3′ end	++	IVT	+++	2016	Yes	[31]
10× Genomics	Droplet	3′ end	+++	PCR	++	2017	Yes	[13]
MARS-seq2.0	Plate	3′ end	++	IVT	+++	2019	Yes	[32]
UDA-seq	Droplet	3′ end	++++	PCR	++	2025	Yes	[33]

^a^Unique molecular identifier

^b^The number of plus symbols (+ to ++++) denotes the relative ranking from low to very high

^c^Polymerase chain reaction

^d^In vitro transcription

Furthermore, the sequencing modality itself—short-read versus long-read platforms—constitutes a critical technical consideration. Short-read sequencing (e.g., Illumina) delivers high accuracy and throughput but typically captures only 3′ or 5′ ends, limiting the resolution of full-length transcript isoforms [34]. In contrast, long-read platforms (e.g., PacBio Iso-Seq or Oxford Nanopore) enable complete mRNA coverage, facilitating gene splicing analyses and identifying novel isoforms [35]. This distinction carries significant biological implications for mosquito biology: short-read approaches may overlook functionally relevant splice variants of immune genes or metabolic enzymes that modulate vector competence, whereas long-read methods, despite lower throughput and higher cost, provide essential resolution for dissecting transcriptomic diversity underlying phenotypic plasticity.

### Data analysis

The analysis of raw sequencing data is equally critical, as its quality directly determines the reliability of biological interpretations. A flawed analysis pipeline can readily generate compelling but misleading biological conclusions, such as inferring nonexistent cell types or misattributing differential expression. The standard workflow encompasses read mapping, quality control, normalization, dimensionality reduction, clustering, differential expression analysis, cell type annotation, and enrichment analysis. The absence of a carefully compiled cell type marker database for mosquitoes complicates annotation efforts, often necessitating inference from homologous genes in *Drosophila* and other organisms. It is important to note that a deep understanding of the statistical methods and parameter choices underlying each analytical step is essential for generating reliable biological interpretations.

A recent scRNA-seq analysis of the *An. gambiae* midgut illustrates a representative analytical pipeline [19]. FASTQ files were mapped to the *An. gambiae* PEST genome (VectorBase.org, v.58) using Cell Ranger v.7.0.1. Quality control in Scanpy filtered cells with complexity score < 0.7 or > 30% mitochondrial reads. The count matrix was converted to Seurat using SeuratDisk for downstream analysis. The top 25% variable genes were selected, data scaled, and PCA performed (first 100 PCs). Integration used RPCA (k.weight = 64) to correct batch effects while preserving biological variation. UMAP was calculated (first 56 PCs), and clustering employed Louvain algorithm (resolution = 0.3). Markers were identified using FindAllMarkers (log2FC > 1). Clusters were annotated by cross-referencing Fly Cell Atlas, *D. melanogaster* gut, *Ae. aegypti* gut, and *An. gambiae* hemocyte studies. Pseudobulk differential expression analysis was performed using AggregateExpression and DESeq2, and functional enrichment was conducted using gProfiler.

The selection of analytical tools must align with the experimental design. For instance, the official Cell Ranger software is typically used for 3′-end-tagged libraries from the 10× Chromium platform. In contrast, for full-length transcript sequencing methods such as Smart-Seq2, software such as STAR is required [24, 36]. While a comprehensive discussion of bioinformatics pipelines and tool benchmarking lies beyond the scope of this review, commonly used software packages in scRNA-seq analysis of mosquito are summarized in Table 2.

**Table 2:** scRNA-seq analysis workflow and software in mosquito studies

**Method**	**Software packages (Language/Platform)**	**Example application in mosquito research**	**Function**
Read mapping	CellRanger (Linux) [37]	*An. gambiae* midgut [19]; *An. gambiae* germline cell [38]; *An. gambiae* hemocytes [13]; *Ae. aegypti* midgut [14, 17, 39]; *Ae. aegypti* whole body [16]; *Ae. aegypti* brain [16, 40]; *Ae. aegypti* ventral nerve cord [41]; *Cx. tarsalis* midgut [18]	Mapping sequencing reads to mosquito reference genomes (VectorBase) for gene expression matrix generation
	STAR (Shell) [42]	*An. gambiae* hemocytes [43]	
	STARsolo (Shell) [42]	*Ae. aegypti* midgut and fat body [15]	
	HISAT2 (Linux) [44]	*An. gambiae* hemocytes [45]	
Quality control	Seurat (R) [46]	*An. gambiae* germline cell [38]; *An. gambiae* hemocytes [13, 45]; *Ae. aegypti* midgut [14, 15, 17]; *Ae. aegypti* ventral nerve cord [41]; *Ae. aegypti* fat body [15]; *Ae. aegypti* brain [40]; *Cx. tarsalis* midgut [18]	Excluding low-quality cells and noisy genes generated during sequencing
	Scanpy (Python) [47]	*An. gambiae* midgut [19]; *Ae. aegypti* midgut [39]; *Ae. aegypti* whole body [16]	
	DoubletFinder (R) [48]	*Ae. aegypti* ventral nerve cord [41]; *Ae. aegypti* midgut [39]	
	Scrublet (Python) [49]	*Ae. aegypti* whole body [16]	
	scDblFinder (R) [50]	*An. gambiae* midgut [19]; *Cx. tarsalis* midgut [18]	
	singleCellTK (R) [51]	*Cx. tarsalis* midgut [18]	
	DecontX (R) [52]	*Cx. tarsalis* midgut [18]	
Normalization; data integration; visualization and clustering	Seurat (R) [46]	*An. gambiae* germline cell [38]; *An. gambiae* midgut [19]; *An. gambiae* hemocytes [13, 45]; *Ae. aegypti* midgut [14, 15, 17]; *Ae. aegypti* ventral nerve cord [41]; *Ae. aegypti* fat body [15]; *Ae. aegypti* brain [40]; *Cx. tarsalis* midgut [18]	Data normalization; integration (CCA^a^, RPCA^b^, Harmony); cell visualization and clustering through linear and nonlinear dimensionality reduction (PCA^c^, *t*-SNE^d^, UMAP^e^);
	Scanpy (Python) [47]	*Ae. aegypti* midgut [39]; *Ae. aegypti* whole body[16]	
Differential expression genes	DESeq2 (R) [53]	*An. gambiae* midgut[19]; *An. gambiae* hemocytes [43]; *Cx. tarsalis* midgut [18]	Differential gene expression analysis
	MAST (R) [54]	*Ae. aegypti* whole body [16]	
	Seurat (R)[46]	*An. gambiae* germline cell [38]; *An. gambiae* midgut [19]; *An. gambiae* hemocytes [13, 45]; *Ae. aegypti* midgut [14, 15, 17]; *Ae. aegypti* ventral nerve cord [41]; *Ae. aegypti* fat body [15]; *Ae. aegypti* brain [40]; *Cx. tarsalis* midgut [18]	
	Scanpy (Python) [47]	*Ae. aegypti* midgut [39]	
Enrichment analysis	topGO (R) [55]	*An. gambiae* hemocytes [43]	Detecting significantly overrepresented pathways/functions in a gene list using databases such as GO^f^ and KEGG^g^
	gProfiler (R) [56]	*An. gambiae* midgut [19]; *Ae. aegypti* midgut [14]	
	PANTHER (Python) [57]	*An. gambiae* germline cell [38]	
	ShinyGO (R) [58]	*Cx. tarsalis* midgut [18]	
Cell type annotation	Seurat (R) [46]	*An. gambiae* germline cell [38]; *An. gambiae* midgut [19]; *An. gambiae* hemocytes [13, 45]; *Ae. aegypti* midgut [14, 15, 17]; *Ae. aegypti* ventral nerve cord [41]; *Ae. aegypti* fat body [15]; *Ae. aegypti* brain [40]; *Cx. tarsalis* midgut [18]	Manual curation based on canonical marker genes and VectorBase
	Scanpy (Python) [47]	*Ae. aegypti* midgut [39]; *Ae. aegypti* whole body [16]	

^a^Canonical correlation analysis

^b^Reciprocal principal component analysis

^c^Principal component analysis

^d^*t*-distributed stochastic neighbor embedding

^e^Uniform manifold approximation and projection

^f^Gene Ontology

^g^Kyoto Encyclopedia of Genes and Genomes

### Research progress of scRNA-seq in mosquito research

Progress in combating mosquito-borne diseases has long been constrained by a limited understanding of vector biology at a fine cellular level. Traditional approaches, often relying on inferences from model organisms such as *Drosophila*, failed to elucidate the cellular and molecular mechanisms underlying unique mosquito behaviors (e.g., blood-feeding) and their susceptibility to pathogens. This section reviews key discoveries in mosquito biology driven by the application of scRNA-seq.

### Construction of mosquito cell atlases

A fundamental goal of cellular biology involves the systematic creation of comprehensive cell type atlases. In mosquitoes, this atlas was historically sparse and reliant on morphological criteria—an approach that fails to distinguish functionally distinct yet morphologically similar cells or to identify rare but critical cell populations. ScRNA-seq has refined morphological cell types into transcriptionally distinct subtypes and uncovered previously undescribed cell populations.

#### Redefinition of cell subtypes

The power of scRNA-seq to unbiasedly redefine cellular subtypes is vividly demonstrated in the immune and digestive systems [13, 14]. In *An. gambiae* hemocytes, the classic classification of granulocytes, oenocytoids, and prohemocytes has been exploded into a functionally multilevel system [59]. Granulocytes, once considered a uniform cell type, have now been discovered to comprise several subtypes, including conventional, proliferative, antimicrobial, and phagocytic, as well as megacytes—a subset believed to coordinate hemocyte differentiation factor (HDF)-mediated immune responses [13, 45]. Notably, megacytes were not detected in *Ae. aegypti* [13], a finding that reflects distinct cellular lineages among different vector species rather than a technical artifact. This observation challenges the traditional view of a universal immune cell lineage in insects and provides a cellular basis for developing targeted control strategies designed for specific mosquito vector species.

Similarly, the midgut epithelium, once broadly divided into enterocytes (ECs), enteroendocrine cells (EEs), and stem/progenitor cells, is revealed as a complex network of specialized modules. ECs and EC-like cells are divided into five subtypes enriched for distinct digestive enzymes (e.g., trypsin and chymotrypsin) or immune factors, indicating a functional specialization for nutrient processing and pathogen surveillance [17]. EEs, the sensory cells of the gut, are divided into three subtypes specializing in different neuropeptide signals or chemosensory pathways [14]. This more detailed view transforms our understanding of the midgut from a simple nutrient-absorbing tube to a tightly regulated, compartmentalized organ where digestion, immunity, and interorgan signaling are partitioned at the cellular level.

#### Discovery of novel cell populations

ScRNA-seq has also revealed novel cell populations that fall outside existing classification schemes. A prime example is the identification of fat-body-yolk cells (FYCs) in fat bodies of blood-fed *Ae. aegypti* [15]. This population defies conventional classification by coexpressing markers of fat body (metabolic) and ovarian (reproductive) tissues, alongside stem cell genes (*klu*, *Delta*) and eggshell formation genes (*Nasrat*). Its persistent presence post blood-meal, even in the absence of oviposition sites, suggests a novel poised state dedicated to maintaining reproductive readiness—a potential adaptation to unpredictable breeding opportunities. While FYCs exemplify the discovery potential of scRNA-seq, they also emphasize a critical caution: transcriptional identity does not equate to validated function. The hypothesized roles of FYCs in reproductive regulation, lipid transport, resource reallocation, and defense [60–63] remain compelling yet untested.

Similarly, cell atlases have been established in more tissues such as the salivary glands, testes, and nervous system [16, 38, 41]. Detailed cell types and marker genes of *Ae. aegypti* and *An. gambiae* are presented in Tables 3 and 4. Although several newly identified cell populations (e.g., salivary gland cells highly expressing *CCC2* and *GPRNPY6*) still require functional validation owing to the absence of definitive marker genes [16], these efforts have laid the foundation for a comprehensive single-cell atlas of mosquitoes. Future efforts to unravel the cell atlases across more mosquito species and tissues will provide a critical reference for deciphering cell-type-specific mechanisms governing vector competence, reproductive biology, and chemosensory behaviors and hold great potential for identifying novel targets for next-generation insecticides, gene-drive strategies, and transmission-blocking interventions.

**Table 3:** Cell type marker genes of *Ae. aegypti* reported in previous studies

**Cell type**	**Marker gene name**	**AAEL ID**	**References**
Midgut			
ISC/EB	*Delta*	AAEL025606	[14]
ISC/EB	*Klu*	AAEL013544	[14]
Cardia	*Gambicin*	AAEL004522	[14]
EC-like	*SP24D*	AAEL008769	[14]
EC	*Nubbin*	AAEL017445	[14]
EE	*Prospero*	AAEL002769	[14]
EE	*NPF*	AAEL002733	[14]
Visceral muscle	*Actin*	AAEL001673	[14]
Fat body	*ApoLp-III*	AAEL008789	[13]
Hemocytes	*LRIM16*	AAEL008658	[13]
Brain			
Glia	*repo*	AAEL027131	[40]
Astrocyte	*wun2*	AAEL007322	[40]
Kenyon cells	*eyeless*	AAEL002321	[40]
Ventral projection neurons	*Lim 1*	AAEL019457	[40]
Monoaminergic neurons	*Vmat*	AAEL026276	[40]
Medulla neurons	*hth*	AAEL011643	[40]
Proximal medulla neurons	*Lim 3*	AAEL007120	[40]
Projection neurons	*ct*	AAEL019970	[40]
Neuron, ChAT	*ChAT*	AAEL010471	[16]
Neuron, Vglut	*Vglut*	AAEL001867	[16]
Neuron, Gad1	*Gad1*	AAEL011981	[16]
Clock cells	*Pdf*	AAEL001754	[16]
Neuron, mechanosensory	*nompC*	AAEL019818	[16]
Neuron, dopamine	*DAT*	AAEL024732	[16]
Salivary gland			
Neurons, GPRNPY6, GPRALS3	*GPRNPY6*	AAEL017005	[16]
Muscle	*troponin T*	AAEL002417	[16]
Distal–lateral lobes	*venom allergen 3*	AAEL000793	[16]
Medial lobes	*CTL16*	AAEL000533	[16]
Proximal–lateral lobes	*MAL1*	AAEL009524	[16]
Hemocytes	*NimB2*	AAEL019650	[16]
Fat tissue	*FAS1*	AAEL001194	[16]
Glia	*repo*	AAEL027131	[16]
Testes			
Vas deferens	*tsl*	AAEL010469	[16]
Fat tissue	*FAS1*	AAEL001194	[16]
Hemocytes	*NimB2*	AAEL019650	[16]
Mid-cyst cells	*sona*	AAEL019697	[16]
Early cyst cells	*FOXF*	AAEL009031	[16]
Late cyst cells	*GPRGRK*	AAEL004777	[16]
Testes epithelium	*5-HT7*	AAEL027242	[16]
Early germline spermatogonia	*vas*	AAEL004978	[16]
Early primary spermatocytes	*betaTub85D*	AAEL019894	[16]
Later spermatocytes	*S-LAP*	AAEL000108	[16]
Spermatids	*Orco*	AAEL005776	[16]
Fat body			
Trophocytes	*Vg*	AAEL006126	[15]
Oenocytes	*AaFAS3*	AAEL022506	[15]
Fat-body-yolk cells	*Nasrat*	AAEL008829	[15]
Tracheoblast-like cells	*nell1*	AAEL005131	[15]
Hemocytes	*NimB2*	AAEL019650	[15]
Muscle cells	*Tm1*	AAEL002759	[15]
Epidermal cells	*Lcp65Ad*	AAEL013515	[15]
Neuroendocrine cells	*Appl*	AAEL005895	[15]

**Table 4:** Cell type marker genes of *An. gambiae* reported in previous studies

**Cell type**	**Marker gene name**	**AGAP ID**	**References**
Hemocytes			
Oenocytoids	*PPO4*	AGAP004981	[13]
Prohemocytes	*SPARK*	AGAP000035	[13]
Granulocytes	*LysI*	AGAP011119	[45]
Dividing granulocytes	*Cycl-B*	AGAP004962	[13]
Megacytes	*TM7318*	AGAP007318	[13]
Antimicrobial granulocytes	*Lysoz-C1*	AGAP007347	[13]
Phagocytic granulocytes	*FBN10*	AGAP011230	[45]
Fat body	*Gelsolin*	AGAP011369	[13]
Muscle	*MyosL Ch5*	AGAP001622	[13]
Germ cells			
Hub cells	*Fas3*	AGAP029564	[38]
Germline stem cells	*squid*	AGAP000399	[38]
Primary spermatogonia	*Aurora kinase*	AGAP007855	[38]
Late spermatogonia/primary spermatocytes	*argonaute 3*	AGAP008862	[38]
Spermatocytes	*LOC1280320*	AGAP012394	[38]
Late spermatocytes/early spermatids	*elf4E-5*	AGAP007172	[38]
Late spermatids	*protamine*	AGAP028569	[38]
Mature spermatozoa	*Dnah3*	AGAP007675	[38]

#### Technical variations and interpretation challenges

The construction of cell atlases is strongly influenced by technical choices, as demonstrated by discrepancies between different studies. For instance, 10× Chromium successfully captured the rare megacyte subpopulation (0.5% abundance) in hemocytes owing to its high throughput [13], whereas Smart-Seq2, despite lower throughput, identified phagocytic granulocytes through its greater sensitivity [45]. Both results were validated by whole-mount in situ hybridization and RNA fluorescence in situ hybridization, confirming that differences stem from technical characteristics rather than false positives. Specifically, 10× Chromium uses high-throughput droplet‑based barcoding to profile tens of thousands of cells, enabling rare subpopulation detection; Smart‑Seq2 employs full‑length complementary DNA (cDNA) amplification and low‑input compatibility, providing higher sensitivity to low‑abundance transcripts, allowing it to identify cell types that 10× Chromium may fail to detect owing to lower transcript sensitivity. Similarly, variations in midgut EC subtype numbers between studies may reflect differences in dissociation methods (whole cell versus nucleus), sampling timepoints, or analytical clustering parameters [14, 17].

These examples revealed a core challenge: distinguishing a robust biological discovery from a technical artifact or an over-interpreted computational clustering result. Therefore, we emphasize that the interpretation of scRNA-seq results should be rigorous to ensure reproducibility. This can be implemented through: (1) adherence to Minimum Information about a Single-Cell Experiment (minSCe) guidelines, which describe a single-cell sequencing experiment with a few additions and changes to the Minimum Information about a Microarray Experiment (MIAME) and Minimum Information about a High-Throughput Nucleotide Sequencing Experiment (MINSEQE) experiment model, capturing information describing each component and linking it to the relevant protocols [64]; (2) explicit documentation of technical parameters (e.g., platform, dissociation protocol, sequencing depth) and computational workflows (including software versions, algorithmic parameters, and code repositories); and (3) validation of novel cell types using methods such as RNA fluorescence in situ hybridization or protein staining. These will ensure that the established mosquito cell atlases serve as a robust foundation for future mechanistic studies, rather than a collection of transient computational artifacts.

### Elucidating biological processes

The construction of cell atlases provides the necessary framework for elucidating dynamic biological processes. Through differential gene expression and functional enrichment analyses for distinct cell populations, scRNA-seq extends beyond static cell classification to the dissection of temporal- and condition-specific transcriptional dynamics. These approaches have been instrumental in elucidating the cellular and molecular mechanisms underlying key physiological phenomena in mosquitoes, such as post-blood-meal responses, sexual dimorphism, and receptor coexpression patterns.

#### Post-blood-meal transcriptional remodeling

The blood meal triggers a multiorgan physiological process essential for reproduction. For instance, scRNA-seq of the midgut reveals that three nascent enterocyte clusters emerged within 1 day post-blood-meal (dpbm), increasing from 0.4% to 52.2% [14]. Given the inability to generate massive new cells in this short timeframe, the origin of these cells remains unclear, with transdifferentiation from existing ECs or enteroblasts proposed as a plausible hypothesis, highlighting a knowledge gap in midgut epithelial plasticity. Interestingly, while metabolic pathways are broadly upregulated, key digestive enzyme genes (e.g., *trypsins*) are downregulated in certain EC subsets, suggesting a complex, nonuniform regulation of digestion that warrants further investigation.

Concomitant changes occur in the fat body. During 2–7 dpbm, the proportion of trophocyte 1 declined, while the expression of trehalose synthesis genes (*Tps1*, *Tpp*) and immune regulators (*Vago-like*) was upregulated in trophocyte 2 and 3, indicating a coupling of metabolic reprogramming and immune activation [15, 65]. Concurrently, the proportion of hemocytes doubled at 7 dpbm, with granulocytes highly expressing immune and cuticle genes, suggesting specialization toward abdominal tissues and participation in local immune responses. Additionally, FYCs stably expressed eggshell formation genes (*Nasrat*) and PIWI pathway genes during this period, maintaining reproductive readiness without oviposition [15, 60, 63].

Neural tissues also participate in this systemic response. SnRNA-seq of the brain revealed the most remarkable transcriptomic changes in glial cells at 3 h post blood-meal, including the upregulation of steroid hormone receptors (*E75*, *EcR*) and circadian clock genes (*PER*), implicating glial cells as potential regulators of the post-blood-meal behavioral and physiological switch [16].

Collectively, these studies revealed a coordinated and modular post-blood-meal response, where distinct cell types within and across organs perform specialized roles in metabolism, immunity, and reproduction. Furthermore, key unresolved questions include the intercellular signaling mechanisms that synchronize this response and the functional validation of the proposed cellular plasticity events.

#### Cellular basis of sexual dimorphism

Sexual dimorphism in mosquitoes extends beyond morphology to encompass behavior and physiology. ScRNA-seq enables a systematic comparison of cellular composition and gene expression between sexes at single-cell resolution.

In the brain of *Ae. aegypti*, compositional differences are evident. Female brains are enriched for Kenyon cells 2, dopaminergic neurons, and projection neurons, while male brains contain higher proportions of specific glial, proximal medulla neurons, and Kenyon cells 3 [40]. These differences are paralleled by sex-biased gene expression: males show higher expression of the sex determination factor *Nix* and neuropeptide Y receptors, whereas females highly expressed dipeptidyl peptidases and signal receptor *Pka-R1* [16, 40]. Notably, the sex determination gene *doublesex* (*dsx*) is more highly expressed in glial cells than neurons, suggesting glial cells may play an underappreciated role in establishing or maintaining sexual identity [16].

Dimorphism is also remarkable in peripheral sensory organs. Male-specific *ppk317*^+^ epithelial-like cells in antennae and female-specific *Ir7e*^+^ neurons in the proboscis have been identified, providing a cellular correlate for sex-specific behaviors such as host-seeking and mating [16].

Together, these findings demonstrated that sexual dimorphism likely arises from sex-specific abundance and regulation of particular cell subpopulations rather than global transcriptional differences.

#### Receptor coexpression and sensory coding

SnRNA-seq analysis of *Ae. aegypti* sensory neurons has systematically revealed coexpression patterns among receptors from different gene families, thereby challenging the conventional “one neuron–one receptor” rule.

In olfactory organs, the receptor *Ir25a* is frequently coexpressed with odorant receptors (ORs), with both *Orco* and *Ir25a* detected together in more than 10% of neurons, revealing flexibility in receptor combinations within the olfactory coding framework [66]. In gustatory tarsi, the low-salt sensor gene *ppk301* is coexpressed with sweet receptor gene *Gr7/Gr9*, and several gustatory neurons also express the thermosensor receptor *TrpA1*, suggesting integration of taste and temperature receptors at the cellular level [16].

These findings significantly expand the theoretical coding capacity of the mosquito sensory system. However, it is critical to distinguish transcriptional coexpression from functional interaction. The physiological consequence of coexpression—whether it enables ligand cross-talk, modulates sensitivity, or broadens tuning—remains a subject for future electrophysiological and behavioral studies.

### Decoding host–pathogen interactions

A core goal in mosquito biology is to understand the cellular and molecular interactions between mosquitoes and the pathogens they transmit. Bulk RNA-seq studies have characterized tissue-level transcriptional responses to infection. For instance, Dong et al. detected the upregulation of immune-related genes in chikungunya virus-infected *Ae. aegypti* midguts [67]. However, this approach cannot resolve which specific cell types mediate these responses or harbor the highest pathogen loads. ScRNA-seq addresses this limitation by profiling host and pathogen transcriptomes at single-cell resolution, enabling the identification of susceptible cell types and cell-type-specific host factors critical for pathogen replication or restriction.

#### Technical adaptation for pathogen detection

Mosquito-borne disease investigations frequently involve multiple organisms—including the vector, pathogen, and microbiome—yet conventional scRNA-seq approaches often filter out nonhost reads as “contamination” during quality control, discarding valuable pathogen and microbial transcriptional information. ScRNA-seq strategies that simultaneously profile host and pathogen transcriptomes from the same cells are essential to capture these multiorganism interactions. A primary challenge of scRNA-seq in studying flavivirus-mosquito interactions is capturing viral RNAs, which often lack polyadenylated tails. The development of 5′ capture scRNA-seq protocols, compatible with large-scale microfluidic platforms, has been pivotal in overcoming this barrier, enabling the precise association of flavivirus RNA with specific host cells [68]. For example, this approach successfully captured ZIKV transcripts in *Ae. aegypti* midgut cells [17].

#### Cellular tropism of mosquito-borne pathogens

Emerging evidence from scRNA-seq studies has revealed that pathogens exhibit remarkable cell tropism within mosquito tissues. To date, only three studies have directly investigated host–pathogen interactions in mosquitoes at single‑cell resolution, each focusing on a different major vector species. For flaviviruses, 5′ capture scRNA-seq in *Ae. aegypti* midguts revealed that ZIKV preferentially targets EEs and ECs [17]. Similarly, West Nile virus (WNV) in *Cx. tarsalis* midguts was found at the highest load within EEs [18]. These findings suggest that EEs may exhibit a specific and conserved susceptibility to flaviviruses, potentially owing to their specialized signaling and secretory functions which could be exploited by the viruses. Interestingly, within WNV-infected midguts, the intestinal stem cell/enteroblast population maintained the lowest viral RNA levels [18], hinting at an intrinsic resistance mechanism that merits further exploration.

In studies of *Plasmodium*-infected *An. gambiae*, dual scRNA-seq capturing both host and parasite transcriptomes revealed a specific interaction preference: ookinetes predominantly interact with progenitor cells during midgut epithelium traversal [19]. This interaction appears evolutionarily conserved across *Anopheles* species and *Plasmodium* *falciparum* isolates, indicating that progenitor cells may present a conserved interaction site for parasite invasion, representing a potential target for transmission-blocking interventions.

#### Dissecting anti-pathogen immune responses

ScRNA-seq extends beyond identifying infected cells to reveal mosquito immune responses at the cellular level. In ZIKV-infected *Ae. aegypti* midguts, differential expression analysis indicated that ECs highly expressed the antimicrobial peptides such as *Gambicin*, suggesting a direct antiviral defense [17]. In contrast, a study of WNV-infected *Cx. tarsalis* found that while intracellular immune gene expression correlated with viral load in infected cells, there was no broad, tissue-level immune activation, and sessile hemocyte numbers in the midgut did not change significantly [18]. This latter finding challenges the traditional view that the midgut is a key site for innate immune activation and may partially explain the high vector competence of certain species.

The power of scRNA-seq to pinpoint key cellular regulators of immunity is exemplified in malaria vector research. Previous work showed that a hemocyte differentiation factor (HDF) enhances anti-*Plasmodium* immunity [69]. ScRNA-seq resolved the hemocyte landscape and identified the *LL3* gene as highly expressed in a rare megacyte subtype. Functional RNAi demonstrated that *LL3* is essential for the HDF-mediated response, establishing megacytes as a critical regulatory node and *LL3* as a candidate intervention target [13, 70]. This study illustrates how scRNA-seq can bridge population-level observations (HDF effect) with specific cellular mechanisms (megacyte function).

#### From observation to intervention

The true value of scRNA-seq in host–pathogen studies lies in its ability to generate mechanistic, testable hypotheses. The enrichment of *apolipophorin III* in ZIKV-infected EEs led to the hypothesis that this lipid transport protein facilitates infection. RNAi confirmed its role in supporting ZIKV replication, validating it as a potential target [17]. Similarly, the discovery of megacytes and *LL3* has prompted investigations into the plasticity of hemocyte differentiation as a target for enhancing antiparasite immunity [13, 70].

Future research should utilize the dynamic potential of scRNA-seq. Time-course studies tracking transcriptional changes in key immune cells post infection can reveal the dynamics of immune responses. Furthermore, expanding the use of dual scRNA-seq to other pathogen–vector pairs will allow systematic mapping of interaction networks at the single-cell level.

## Conclusions

ScRNA-seq has enabled the dissection of mosquito tissues into their constituent cell types, moving the field from tissue-level averages toward a resolved view of cellular heterogeneity, yet significant challenges remain.

Current efforts are constrained by methodological and biological gaps. The reliance on orthology-based annotation with *Drosophila* markers may lead to the misannotation of mosquito-specific cell types. Furthermore, cell atlases remain incomplete across species and organs (e.g., ovary and compound eyes), which currently limits the ability to develop a systematic, organism-wide perspective. Most importantly, the field has generated a substantial number of transcriptional hypotheses (e.g., cellular transdifferentiation and receptor coexpression) [14, 16], which require rigorous functional validation via the integration of both classical and modern genetic tools.

Future progress in this field will rely on the integration and application of multidisciplinary technologies. The foremost priority remains the expansion of cell atlases to encompass diverse mosquito species and tissue types. It is essential to integrate spatial transcriptomics and single-cell multiomics technologies, which will enable the precise identification of cell types within their natural tissue structure and unravel related gene regulatory networks. Specifically, spatial transcriptomics can map the spatial distribution of viral or parasitic infection foci within midgut epithelium and salivary glands, revealing how pathogen load correlates with regional cellular composition and microenvironmental gradients. Furthermore, the integration of spatial transcriptomics with single-cell epigenomic profiling (e.g., scATAC-seq) and proteomic analysis will enable the construction of spatially resolved gene regulatory networks, linking chromatin accessibility states and protein expression patterns to transcriptional phenotypes within distinct tissue microenvironments. Ultimately, these multidimensional datasets will be used to build predictive models of mosquito–pathogen interactions, identify key regulatory molecules and potential intervention targets, and validate these findings via temporally dynamic and perturbation-based experiments.

In summary, scRNA‑seq is fundamentally reshaping our understanding of mosquito biology. Its power lies not only in mapping cell atlases but also in paving the way for innovative strategies to control mosquito‑borne diseases. By systematically addressing current challenges, scRNA‑seq is maturing from a powerful discovery tool into a foundational platform for developing targeted vector‑control interventions. Ultimately, translating cutting‑edge single‑cell insights into effective public health measures will remain the ultimate challenge and objective for this field.

## Data Availability

Data supporting the main conclusions of this study are included in the manuscript.

## Abbreviations

CHIKV: Chikungunya virus
dpbm: Day post blood-meal
dsx: Doublesex
ECs: Enterocytes
EEs: Enteroendocrine cells
FYCs: Fat-body-yolk cells
HDF: Hemocyte differentiation factor
ORs: Odorant receptors
PCA: Principal component analysis
PCR: Polymerase chain reaction
RPCA: Reciprocal principal component analysis
RNAi: RNA interference
RNA-FISH: RNA fluorescence in situ hybridization
scRNA-seq: Single-cell RNA sequencing
snRNA-seq: Single-nucleus RNA sequencing
scATAC-seq: Single-cell assay for transposase-accessible chromatin using sequencing
UMAP: Uniform manifold approximation and projection
UMI: Unique molecular identifier
WNV: West Nile virus
ZIKV: Zika virus
